# Neurologic features in hospitalized patients with COVID-19: a prospective cohort in a catalan hospital

**DOI:** 10.1007/s10072-025-08031-y

**Published:** 2025-02-14

**Authors:** Oriol Barrachina-Esteve, A. Anguita, A. Reverter, J. Espinosa, C. Lafuente, M. Rubio-Roy, M. Crosas, C. Vila-Sala, C. Acero, M. Navarro, D. Cánovas, G. Ribera, M. Jodar, J. Estela

**Affiliations:** 1 Department of Neurology, Parc Taulí University Hospital, Parc Taulí Research and Innovation Institute Foundation (I3PT), Universitat Autònoma de Barcelona, Sabadell, Spain; 2 Department of Neurology, Manacor Hospital, Manacor, Mallorca Spain; 3 Department of Neurology, Sant Joan de Déu Hospital, Althaia Foundation, Manresa, Spain; 4 Department of Ophthalmology, Parc Taulí University Hospital, Parc Taulí Research and Innovation Institute Foundation (I3PT), Universitat Autònoma de Barcelona, Sabadell, Spain; 5 Department of Infectious Diseases, Parc Taulí University Hospital, Parc Taulí Research and Innovation Institute Foundation (I3PT), Universitat Autònoma de Barcelona, Sabadell, Spain; 6 Department of Clinical and Health Psychology, Universitat Autònoma de Barcelona, Barcelona, Spain; 7 Centre for Biomedical Research in the Mental Health Network (CIBERSAM), National Institute of Health Carlos III, Madrid, Spain

**Keywords:** Neurovirology, COVID-19, Clinical neurology, Cognitive disorders

## Abstract

**Objectives:**

To study the prevalence and timing of neurological manifestations, including cognitive involvement, in patients hospitalized for Coronavirus disease 2019 (COVID-19). To analyze the pathogenic mechanisms and any association they have with disease severity.

**Methods:**

Longitudinal cohort study with prospective follow-up of patients who required hospitalization. Patients under 65 who had no pre-existing cognitive impairment and did not require an ICU stay were evaluated 3 and 12 months after discharge using a battery of neuropsychological tests.

**Results:**

Of 205 patients hospitalized for COVID-19, 153 (74.6%) presented with neurological manifestations. The most frequent were myalgia (32.7%), headache (31.7%), dysgeusia (29.2%), and anosmia (24.9%). Patients with more severe illness at the time of hospitalization presented fewer neurological manifestations. Of the 62 patients who underwent neuropsychological examination 3 months after discharge, 22.6% had impaired attention, 19.4% impaired working memory, 16.1% impaired learning and retrieval, 9.7% impaired executive functions, and 8.2% impaired processing speed. Patients with anosmia also presented with more headache (OR 5.45; p < 0.001) and greater risk of working memory impairment (OR 5.87; p 0.03). At follow-up 12 months after hospital discharge, 14.3% of patients still showed impaired attention, 2.4% impaired working memory, 2.5% impaired executive functions, and 2.5% impaired processing speed.

**Discussion:**

Neurological manifestations are common in patients hospitalized for COVID-19 regardless of severity. The high prevalence of anosmia and its association with headache and working memory impairment at 3 months, suggest potential direct or indirect damage to the prefrontal cortex via invasion of the olfactory bulb by COVID-19.

**Supplementary Information:**

The online version contains supplementary material available at 10.1007/s10072-025-08031-y.

## Introduction

During the COVID-19 pandemic, the disease was claimed to be associated with a high rate of neurological complications. This was initially supported by general clinical series and retrospective studies that described myalgia, headache, dizziness, delirium, and epileptic seizures with an incidence of 25–57%, as well as anosmia and dysgeusia in 5% of patients [1–5]. Nevertheless, a retrospective study on 917 patients in China reported a rate of less than 5% of new-onset critical neurological events after ruling out non-specific manifestations [6].

The real incidence is unclear since most of these studies are retrospective or collected data through electronic health records. Nor is it clear whether neurological complications are caused by COVID-19 directly, indirectly (immune-mediated or hypercoagulable), or non-specifically in the context of severe or prolonged disease. Two prospective studies in which neurologists assessed only patients with neurological manifestations reported neurological disorders in 3.5% (1,865/52,759) [7] and 13.5% (606/4,491) [8] of hospitalized patients with COVID-19. An individual patient data meta-analysis (83 studies, 1979 patients) reported encephalopathy (49%) and cerebrovascular events (26%) as the most common diagnoses [9].

We conducted a prospective observational study of the neurological manifestations of patients hospitalized for COVID-19. We also studied the cognitive function of the subgroup of patients under 65 with no pre-existing cognitive impairment and who did not require an ICU stay. The Neurology department followed all patients to analyze the prevalence, timing, and possible causal relationship with SARS-CoV-2 infection. We also analyzed whether there was any relationship between these manifestations and the severity of the disease.

## Materials and methods

This is a longitudinal cohort study with prospective follow-up of patients with COVID-19 hospitalized at Parc Taulí University Hospital (serving a population of 500,000 inhabitants) to examine the neurological manifestations they present with.

Patient recruitment ran from 3 April to 24 May 2020 and included all patients over 18 who tested positive for SARS-CoV-2 on a PCR test taken via nasopharyngeal swab and who required hospitalization due to their comorbidities, prognostic index (CURB-65 > 2), or severity criteria.

Exclusion criteria included patients with limiting cognitive impairment (GDS > 4), intellectual disabilities, and being unable to collaborate in their medical history (limiting neurological or psychiatric sequelae).

Patients were included in the study within days of testing positive for COVID-19. A neurologist from the research team conducted an exhaustive protocol-based medical history and neurological examination and completed a data collection form. We conducted prospective follow-up of patients for one month, weekly while hospitalized and by phone after being discharged.

All neurological symptoms were recorded and temporarily classified based on whether they appeared before hospitalization (early symptoms), during the acute phase of hospitalization, or during the recovery phase (> 15 days after hospitalization, except for long-stay ICU patients). The research team’s neurologists evaluated and studied patients with neurological manifestations to diagnose them following standard clinical practice. The team’s ophthalmologist evaluated all ocular manifestations.

After data collection, the research team categorized the relationship between each neurological diagnosis and SARS-CoV-2 infection: direct (invasion), indirect (immunological or hypercoagulability), or non-specific (in the context of severe disease or prolonged hospitalization).

Lastly, patients under 65 with no pre-existing cognitive impairment and who did not require an ICU stay [10, 11] completed a battery of 6 neuropsychological tests providing 15 cognitive measures 3 and 12 months after discharge. These cognitive measures were categorized into the following 5 indexes: the attention index, based on the Digit Span Forward subtest of the WAIS Scale-IV; the working memory index, based on the Digit Span Backward subtests of the WAIS Scale-IV; the learning/memory index, based on the learning score, short-term score, and long-term score of the Rey Auditory Verbal Learning Test; the executive functions index, based on the Trail Making Test, Part B, the color and word task of the Stroop test, and the F-A-S Phonemic Verbal Fluency Test; the speed processing index, based on the Trail Making Test, Part A, the digit symbol substitution subtest of the WAIS Scale-IV, and the color and word task of the Stroop test. The raw data from all the neurocognitive tests were converted to z scores (mean = 0, SD = 1) depending on the normative data and averaged to create the previous 5 indexes. The presence of anxiety and/or depression was assessed using the HADS scale. The cut-off points for the HADS-A and HADS-D scales were as follows: normal (0–7); mild (8–10); moderate (11–16); severe (≥ 7) [12].

Patients received pharmacological treatment following hospital protocol, which is based on current evidence and the availability of certain drugs in our health system.

### Statistical analysis

SPSS-25 Statistics was used for all the analyses. An initial descriptive analysis of the study population was conducted, including the mean, median, standard deviation, and quartiles for quantitative variables, and frequencies and percentages for qualitative variables. The main variable was then analyzed by calculating the prevalence of neurological manifestations. Afterwards, the relationship for neurological manifestations was inferred (excluding those with a non-specific relationship with SARS-CoV-2 infection). A univariate analysis was performed to examine the relationship with patient severity at the time of hospitalization (CURB-65, SpO2/FiO2, and need for respiratory support). The Chi-squared test was used for CURB-65 (categorized as < or ≥ 2) and need for respiratory support, with a 95% confidence interval for the odds ratio. The Mann-Whitney U test was used for SpO2/FiO2. The relationship between the various manifestations was also analyzed using the Chi-squared test with a 95% confidence interval for the odds ratio.

To study the cognitive dimensions, the z-scores for age, gender, and education were obtained using the normative data. This allowed us to determine each patient’s deviation from the expected median of all 5 cognitive categories. The cutoff for clinical relevance was performance ≥ 1.5 standard deviations around the mean expected score, and we estimated the frequency of clinically relevant deficit for each cognitive dimension. We explored the relationship between cognitive and psychopathological variables and the neurological manifestations and clinical-analytical variables using bivariate logistic regressions to analyze variables that were significantly related in the final logistic regression model.

## Results

We examined 293 patients hospitalized at our center for eligibility during the inclusion period. A total of 205 patients were included 4 days after testing positive on average. The mean prospective follow-up period was 32 days. Table 1 presents the epidemiological characteristics and Table 2 presents the clinical characteristics of SARS-CoV-2 infection at the time of hospitalization. 

**Table 1:** Baseline characteristics (n 205) aQualitative variables bNon-normal quantitative variables cBody Mass Index dChronic Obstructive Pulmonary Disease eDeep Vein Thrombosis/Superficial Vein Thrombosis/Pulmonary ThromboEmbolism fAngiotensin-Converting Enzyme Inhibitor/Angiotensin Receptor Blocker gModified Rankin Scale for Neurologic Disability hGlobal Deterioration Scale

	*n* (%)^a^, median (P25-P75)^b^
**Demographic data**	
Female	100 (48.8)
Age	70 (57–83)
**Systemic comorbidities**	
Smoker or ex-smoker	54 (26.3)
Alcoholism	8 (3.9)
BMI^c^ >25	134 (65.4)
Hypertension	120 (58.5)
Hypercholesterolemia	77 (37.6)
Diabetes mellitus	51 (24.9)
Atrial fibrillation	44 (21.5)
Chronic kidney disease	35 (17.1)
Coronary heart disease	31 (15.1)
Heart failure	22 (10.7)
COPD^d^	24 (11.7)
Peripheral artery disease	11 (5.4)
DVT/SVT/PTE^e^	11 (5.4)
**Neurologic comorbidities**	
Cerebrovascular disease	24 (11.7)
Lacunar stroke	14 (6.8)
Large vessel occlusion	10 (4.9)
Transient ischemic attack	9 (4.4)
Deep hematoma	1 (0.5)
Encephalitis	2 (1)
Polyneuropathy	2 (1)
**Medication**	
Statins	53 (25.9)
Antiplatelets	37 (18)
Anticoagulants	38 (18.5)
ACEi/ARB^f^	72 (35.1)
**mRankin** ^g^	
0–2	154 (75.1)
3–4	51 (24.9)
**Cognitive impairment**	28 (13.7)
GDS^h^ 2	1 (3.6)
GDS^h^ 3	16 (57.1)
GDS^h^ 4	11 (39.3)

**Table 2:** Clinical characteristics of SARS-CoV2 infection at hospitalization ^a^Qualitative variables ^b^Non-normal quantitative variables ^c^Normal quantitative variables ^d^The n-result is specified for variables with significant lost-to-follow-up ^e^Severity score for Community-Acquired Pneumonia ^f^Brescia-COVID respiratory severity scale

	*n* (%)^a^, median (P25-P75)^b^, mean (deviation)^c^	*n* ^d^		*n* (%)^a^, median (P25-P75)^b^, mean (deviation)^c^			*n* ^d^
**Symptoms**			**Analysis**				
Fever	140 (68.3)		Leukocytes (x10^9^/L)	6.6 (5.12–9.18)			
Cough	113 (55.1)		Lymphocytes (%)	**16.4 (10.15–23.1)**			
Malaise	83 (40.5)		Hemoglobin (g/L)	131 (109–147)			
Dyspnea	69 (33.7)		Platelets (x10^9^/L)	194 (161.5–254)			
Diarrhea	67 (32.7)		Urea (mg/dL)	40 (27-71.8)			
Asthenia	34 (16.6)		Creatinine (mg/dL)	0.9 (0.8–1.3)			
Nausea/vomiting	17 (8.3)		Glycemia (mg/dL)	113 (100–154)			
Anorexia	10 (4.9)		PCR (mg/dL)	6.81 (2.4–10.2)			
Pleuritic pain	9 (4.4)		PT (ratio)	1.2 (1.1–1.3)			
Low-grade fever	9 (4.4)		PTT (ratio)	1 (0.9–1.1)			141
Hypoxic syncope	6 (2.9)		D-dimer (ng/mL)	1008.5 (525–1818)			81
Abdominal pain	2 (1)		AST (U/L)	29 (20–44)			
Mouth ulcers	1 (0.5)		ALT (U/L)	25 (15-43.5)			
Days between symptoms and SARS-CoV2 PCR	5 (2–9)		Fibrinogen	4.5 (3.4–5.5)			
**Vital signs**			LDH (U/L)	266 (204.8–346)			125
Temp (°C)	36.8 (1)		CK (U/L)	48 (29-94.8)			
HR (bpm)	87.3 (17.9)		Ferritin (ng/mL)	626 (316–1290)			140
SBP (mmHg)	131 (24.2)		PaO2 (mmHg)	71 (58.2–97)			81
DBP (mmHg)	72.9 (14.9)		**Chest X-ray**				
RR (breaths/minute)	20 (18–24)	157	Normal	27 (13.2)			
SpO2/FiO2 < 300	23 (11.2)		Unilateral infiltrate	17 (8.3)			
**CURB-65** ^e^			Bilateral infiltrate	161 (78.5)			
0–1	96 (46.8)						
2	81 (39.5)						
3–5	28 (13.7)						
**BCRSS** ^f^							
0	87 (42.4)						
1	78 (38)						
2	29 (14.1)						
3	7 (3.4)						
> o = 4	4 (2)						

At the end of the follow-up, 150 patients had been discharged (73.2%), 46 had died (22.4%), and 10 remained hospitalized for recovery (4.9%). Respiratory support was necessary for 34 patients (16.6%): 6 (2.9%) needed high flow oxygen therapy (HFO), 30 (14.6%) non-invasive mechanical ventilation (NIMV), and 9 (4.4%) invasive mechanical ventilation with orotracheal intubation (IMV and OI).

The mean age of deaths was 80 years, 29 were male and 17 female. Twelve patients had preexisting cognitive decline and 28 had a mRankin ≥ 2. COVID-19 was the cause of death in 43 patients, of which 42 had bilateral pneumonia, 6 acute respiratory distress syndrome (ARDS), 4 pulmonary embolisms, and 1 secondary hemophagocytic lymphohistiocytosis (sHLH). In this group, there were also other comorbidities as the cause of death: 11 patients had heart failure or arrhythmias and 6 had kidney failure. The remaining 3 deaths were attributed to other defined diseases not related to COVID-19 (heart failure, malignant arrhythmias, and kidney failure). There were no deaths caused by neurological diseases that could be related to COVID-19.

Overall, 153 patients (74.6%) presented with neurological manifestations, the majority appearing at the start of the viral infection. The most frequent were myalgia (32.7%), headache (31.7%), dysgeusia (29.2%), anosmia (24.9%), and encephalopathy (22%) (Table 3). After diagnosing each neurological symptom, 25.7% of cases had a non-specific relationship with SARS-CoV-2 (Supplemental Table 1).

**Table 3:** Onset and one-month evolution of neurological manifestations

	Appearance (*n* 205)		Evolution at one month (*n* 159)	
	Total (*n* (%))	Start (*n* (%))	Total assessed (*n*)	Persistence (*n* (%))
Anosmia	51 (24.9)	49 (96.1)	51	5 (9.8)
Dysgeusia	60 (29.2)	58 (96.7)	57	2 (3.5)
Vertigo	2 (1)	2 (100)	2	0 (0)
Headache	65 (31.7)	59 (90.1)	63	12 (19)
Encephalopathy	45 (22)	31 (68.9)	28	1 (3.6)
Tremor	7 (3.4)	0 (0)	7	6 (85.7)
Vision disorders	7 (3.4)	1 (14.3)	7	4 (57.1)
Myalgia	67 (32.7)	64 (95.5)	58	5 (8.6)
Myopathy	16 (7.8)	1 (6.3)	15	11 (73.3)
Neuropathy	10 (4.9)	2 (20)	10	7 (70)

### Absence of dyspnea

Despite requiring oxygen therapy during hospitalization, 52 patients (25.4%) did not report dyspnea. Of these, 46 (88.5%) had bilateral pneumonia, 13 (25%) required respiratory support, and 12 (23.1%) died. Of the deaths, 4 had pre-existing cognitive impairment. The absence of dyspnea is significantly related to increased severity of illness at the time of hospitalization with lower SpO2/FiO2 and CURB-65 ≥ 2. It is also related to the need for respiratory support, although not significantly (Table 4).

**Table 4:** Relationship with severity of the most prevalent neurological manifestations ^a^Median (P25-P75) for SpO2/FiO2, OR CI 95% for CURB-65 and respiratory support ^b^Mann-Whitney for SpO2/FiO2, Pearson’s chi-squared test for CURB-65 and respiratory support

Absence of dyspnea		Median/OR	*p*
SpO2/FiO2	No absence of dyspnea	447.62 (369.23–461.90)	**0.005**
	Absence of dyspnea	421.43 (329.46-452.38)	
CURB-65 ≥ 2		1.96 (1.02–3.78)	**0.041**
Respiratory support		2.1 (0.96–4.56)	0.059
**Anosmia**			
SpO2/FiO2	No anosmia	428.57 (345.33-457.14)	**< 0.001**
	Anosmia	457.14 (442.86-466.67)	
CURB-65 ≥ 2		0.1 (0.04–0.22)	**< 0.001**
Respiratory support		0.92 (0.34–2.17)	0.842
**Dysgeusia**			
SpO2/FiO2	No dysgeusia	428.57 (342.86-457.14)	**< 0.001**
	Dysgeusia	457.14 (442.86-465.48)	
CURB-65 ≥ 2		0.14 (0.07–0.29)	**< 0.001**
Respiratory support		0.70 (0.30–1.66)	0.421
**Headache**			
SpO2/FiO2	No headache	433.33 (342.86-457.14)	**< 0.001**
	Headache	457.14 (428.57-466.67)	
CURB-65 ≥ 2		0.21 (0.11–0.41)	**< 0.001**
Respiratory support		0.72 (0.31–1.71)	0.459
**Encephalopathy**			
SpO2/FiO2	No encephalopathy	442.86 (355.63-459.52)	0.862
	Encephalopathy	440.48 (367.31-459.52)	
CURB-65 ≥ 2		0.87 (0.27–2.81)	0.821
Respiratory support		0.44 (0.06–3.53)	0.428
**Encephalopathy**, **any cause**			
SpO2/FiO2	No encephalopathy	447.62 (369.23–461.90)	**0.03**
	Encephalopathy	385.71 (344.51-457.14)	
CURB-65 ≥ 2		2.33 (1.15–4.7)	**0.017**
Respiratory support		0.725 (0.28–1.88)	0.507
**Myalgia**			
SpO2/FiO2	No myalgia	428.57 (341.07-457.14)	**< 0.001**
	Myalgia	457.14 (429.76-465.48)	
CURB-65 ≥ 2		0.19 (0.1–0.37)	**< 0.001**
Respiratory support		1.46 (0.68–3.13)	0.334

### Anosmia and dysgeusia

A total of 51 patients presented with anosmia. Of these, in 49 (96.1%) it was an early symptom. Two patients presented with anosmia in the recovery phase (at 18 and 27 days of evolution). At the one-month follow-up, anosmia had progressively resolved in 35 patients, improved in 11, and persisted in 5. Sixty patients presented with dysgeusia. Of these, in 58 (96.7%) it was an early symptom. Patients described this symptom as the loss or alteration of taste (often describing a salty, bitter, or metallic taste). At the one-month follow-up, dysgeusia had progressively resolved in 45 patients, improved in 12, and persisted in 3 (Table 3).

Patients with more severe illness at the time of hospitalization, with CURB-65 ≥ 2 and lower SpO2/FiO2, reported significantly less anosmia and dysgeusia (Table 4).

Patients with anosmia presented with significantly more headache (OR 5.45 95% CI 2.75–10.78; *p* < 0.001) and more absence of dyspnea (OR 0.82 95% CI 0.16–0.92; p 0.028).

### Headache

Of the 65 patients who presented with headache, it was attributed to the viral infection in 59 (90.8%). In these cases, it was an early symptom, holocranial, mild to moderate in intensity, oppressive, and intermittent, often in connection with fever. One patient presented with a migraine. Tension headache was diagnosed in 5 patients; in 4 cases it appeared during the recovery phase, unrelated to systemic symptoms, and in 1 case it appeared during the acute phase of hospitalization. In this last case, it was an aggravation in the intensity and frequency of prior chronic tension headaches (Supplemental Table 1).

One month after onset, headache had resolved in 43 patients (68.3%), mostly self-limiting within 7 days, and had improved in 8 patients. It persisted in 12 patients, of which 4 had tension headache, and 8 had headache associated with the viral infection, without evident improvement after the viral infection had resolved (Table 3).

Patients with more severe illness at the time of hospitalization with CURB-65 ≥ 2 and lower SpO2/FiO2 reported less headache associated with SARS-CoV-2 (Table 4).

### Vertigo

Two patients presented with peripheral vertigo as an early symptom that resolved on its own within days, with direct or indirect relationship with the SARS-CoV-2 infection (Table 3, Supplemental Table 1).

### Encephalopathy

Decreased consciousness and/or confusion were reported for 45 patients (22%) (Table 3). Of these, in 33 patients with pre-existing comorbidities (cognitive impairment, kidney failure, etc.) it was related to systemic repercussions of the infection (hypoxia, hypercapnia, hyponatremia, etc.). In 12 cases, encephalopathy associated with viral infection was diagnosed after ruling out systemic causes (Supplemental Table 1). These patients had a mean age of 57 and presented with mild symptoms of bradypsychia, drowsiness and disorientation not attributable to any other cause besides the viral infection itself. At the one-month follow-up, 4 patients had died, and the encephalopathy persisted in 1, and had resolved in 7 (Table 3).

The statistical analysis showed no significant relationship between encephalopathy associated with SARS-CoV-2 and the severity of illness at the time of hospitalization. However, encephalopathy due to any cause was related to CURB-65 ≥ 2 and significantly lower SpO2/FiO2 at the time of hospitalization (Table 4).

**Fig. 1:**
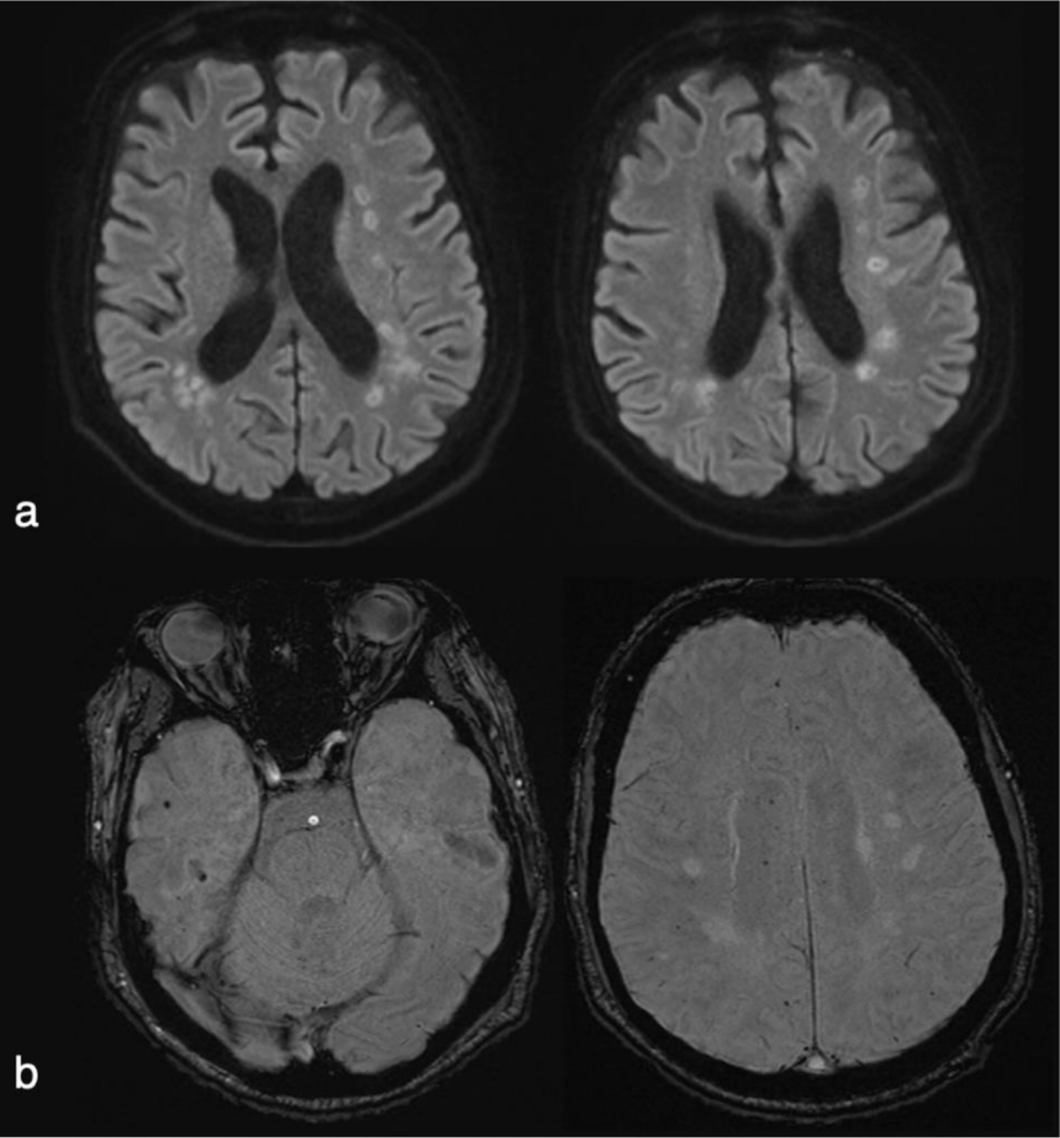
Brain MRI (**a**) DWI sequence: Multiple focal images predominantly periventricular and in both bilateral semioval centers, some of which present with restricted diffusion (not seen but they are hyperintense on T2/FLAIR and hypointense on T1) (**b**) SWI sequence: Hypointense punctiform foci that suggest microhemorrhages diffusely distributed in both supratentorial and infratentorial regions (cerebellar hemispheres and pons), in the corpus callosum and basal ganglia

Multiple diffuse ischemic injuries and microhemorrhages were detected in cranial MRI of a 64year-old patient with acute respiratory distress syndrome (ARDS) and delayed awakening (Fig. 1). This was attributed to extracorporeal membrane oxygenation (Supplemental Table 1).

### Cerebrovascular disease

Two cardioembolic ischemic strokes were diagnosed due to unknown atrial fibrillation neither directly nor indirectly related to COVID-19. The first case was an elderly woman brought in for aphasia and fluctuating consciousness due to an artery of Percheron with a positive PCR test who subsequently developed the disease. In the second case, an aged woman had an infarction in the right temporo-occipital region presenting with left homonymous hemianopsia during the acute phase of the disease (Supplemental Table 1).

### Epilepsy

One focal seizure secondary to chronic parietal lobe stroke neither directly nor indirectly related to COVID-19 was reported. It was an old patient who, during the acute phase of hospitalization, presented with self-limiting symptoms of bradypsychia, dysarthria, and left brachial paresis (Supplemental Table 1).

### Movement disorders

Tremor was assessed in 7 patients (Table 3) neither directly nor indirectly related to COVID-19. In 2 cases it was attributed to aggravated essential tremor in the context of medication and/or hypoxia. In another 2 cases it was attributed to aggravated pre-existing parkinsonism in the context of treatment withdrawal and/or fever. In 3 additional cases, it was iatrogenic due to corticosteroids, also in the context of hypoxia (2) and lopinavir/ritonavir (1). Myoclonus were also observed in 7 patients in the context of hypoxic, toxic, and metabolic encephalopathy, neither directly nor indirectly related to COVID-19 (Supplemental Table 1).

### Vision disorders

Vision disorders were reported in 7 patients (Table 3): self-limiting blurred vision in 3 and myodesopias in 4. Upon evaluation by ophthalmologist, 1 bilateral vitreous detachment, 1 epiretinal membrane, and 1 glaucoma were diagnosed. There were no findings in the remaining cases, and they were attributed to the context of systemic viral infection, directly or indirectly related to COVID-19 (Supplemental Table 1).

### Neuropathy

Ten patients reported symptoms compatible with neuropathy (Table 3). It was not possible to rule out acute polyneuropathy in the context of systemic viral infection in 2 patients with paresthesia and cramping in all four limbs as early symptoms with normal EMG. In the remaining patients, symptoms were neither directly nor indirectly related to COVID-19. In 7 cases, compressive neuropathies were diagnosed in the recovery phase (6 patients requiring a long ICU stay for respiratory support) with involvement of various nerves unilaterally or bilaterally: ulnar (3), external popliteal sciatic (3), lateral femoral cutaneous (2) and internal popliteal sciatic (1). In one of these 7 patients, axonal sensorimotor polyneuropathy of critical illness was also observed. In 1 patient, it was related to chronic lumbar spinal stenosis due to immobilization (Supplemental Table 1).

### Myopathy

Of the 67 patients who reported myalgia, 95.5% did so as an early symptom (Table 3), and it was attributed to the systemic viral infection. In 3 cases it was reported during the recovery phase, after hospital discharge, with no apparent relation to systemic viral infection (Supplemental Table 1). These patients did not have higher CK or LDH than patients without myalgia. At prospective follow-up at one month, myalgia had resolved in 43 patients (74.1%), improved in 10 (17.2%), and persisted in 5 (8.6%) (Table 3).

Myopathy was observed in 16 patients (Table 3), all of whom received hydroxychloroquine, 8 of whom received corticosteroids, and 6 respiratory support. It was diagnosed in 7 cases of critical illness myopathy (patients in critical condition with a mean age of 63) and in 9 cases of disuse muscle atrophy (multipathological patients with a mean age of 84.3) (Supplemental Table 1). In prospective follow-up at one month, myalgia was improving in 4 patients (26.7%) and persisted in the remaining 11 (73.3%) (Table 3).

### Cognitive assessment

Of the 205 patients in the cohort, 143 were excluded from this part of the study due to exclusion criteria. The remaining 62 (30.2%) with the epidemiological and clinical characteristics described in Supplemental Table 2 were neuropsychologically assessed. At follow-up at 12 months, 41 of these patients were reassessed (21 lost to follow-up).

**Fig. 2:**
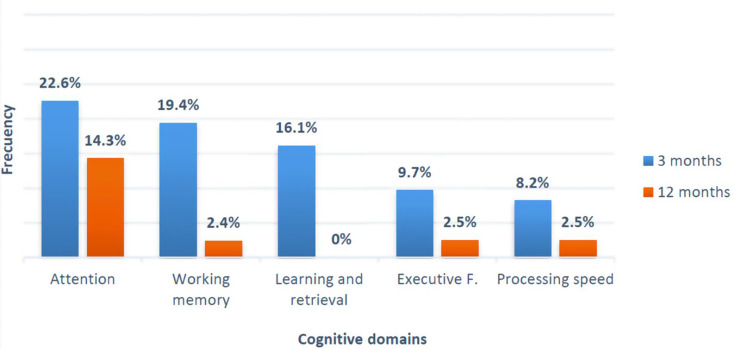
Frequency of cognitive domains impairment at 3 and 12 months

At 3 months after hospital discharge, 22.6% of patients had impaired attention, 19.4% impaired working memory, 16.1% impaired learning and retrieval, 9.7% impaired executive functions, and 8.2% impaired processing speed. At 12 months after hospital discharge, 14.3% of patients showed impaired attention, 2.4% impaired working memory, 2.5% impaired executive functions, and 2.5% impaired processing speed. None had impaired learning and retrieval (Fig. 2).

**Fig. 3:**
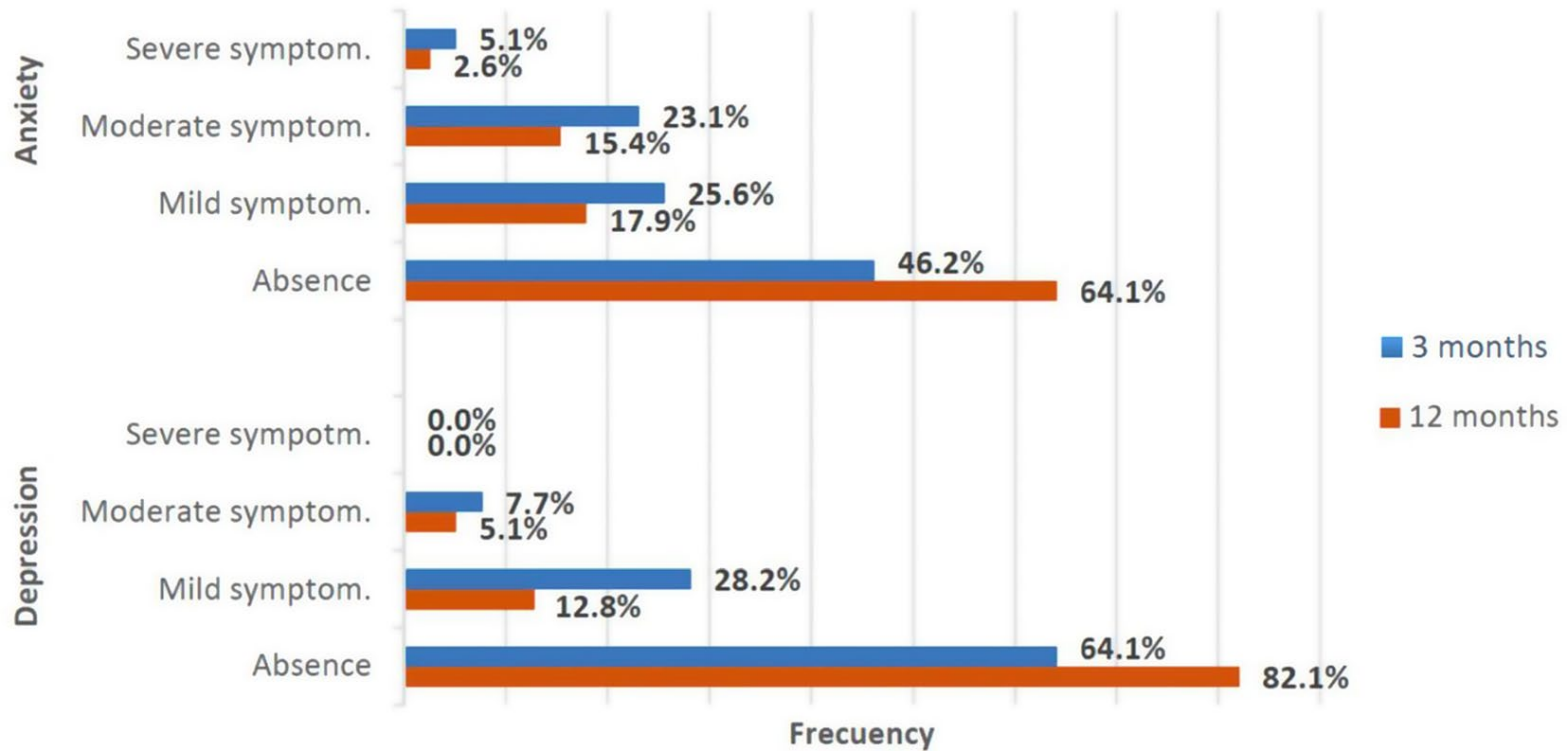
Frequency of affective symptoms at 3 and 12 months

Additionally, 12.6% of patients had anxiety 3 months after hospital discharge and 5.8% did so one year later. 8.9% and 2.8% of the sample had depressive symptoms at 3 and 12 months respectively (Fig. 3). The presence of anosmia (OR = 5.87; *p* = 0.032) and myalgia (OR = 9.37; *p* = 0.039) were statistically related to working memory impairment at 3 months. We found no relationship between any other cognitive dimensions, HADS, or the other clinical and analytical variables analyzed.

## Discussion

Patients hospitalized for COVID-19 commonly present with neurological manifestations (74.6%), generally as early symptoms of the disease. This prevalence is likely higher than that described in similar studies [7, 8] because it is a prospective study with strict clinical follow-up.

SARS-CoV-2 was detected in the brain tissue from autopsies of patients with COVID-19 [13, 14]. It was also detected in neurons of the cerebral cortex [15]. These findings suggest that the neurological symptoms associated with COVID-19 could be the result of direct invasion of the CNS.

One of the most striking phenomena in clinical practice is the absence of dyspnea. This is known as “happy hypoxemia” and has been observed even in the most severe cases. In the Wuhan retrospective cohort, 62.4% of severe cases and 46.3% of patients who were intubated, ventilated or who died did not present with dyspnea [3, 16]. In our study, 25.4% did not report dyspnea despite requiring oxygen therapy during hospitalization. The absence of dyspnea has been considered yet another symptom of the central involvement of the virus [17], even contributing to respiratory failure due to central hypoventilation [16]. Nevertheless, it is more likely due to various pathophysiological mechanisms at the pulmonary level [18], which would support the results of our cohort, in which, paradoxically, the absence of dyspnea is related to less anosmia (supposedly caused by the virus entering the CNS through the olfactory bulb).

The literature reports highly variable rates of anosmia and dysgeusia, ranging from 5% [4, 5] to 80% [19]. These manifestations were very frequent in our clinical series (24.9% and 29.2%, respectively) and are generally among the early symptoms of the disease (96.1% and 96.7%, respectively). It should be noted that lopinavir/ritonavir can infrequently cause dysgeusia (according to the drug fact sheet), although the vast majority of our patients presented with it before hospitalization. This smell and taste disorder appears significantly more frequently than in other viruses involving the upper respiratory tract, such as the flu, and is not accompanied by nasal congestion or rhinitis [20].

Although the pathogenic mechanisms of olfactory dysfunction remain unknown, the initial anosmia in many patients may be related to viral neuroinvasion via transneuronal pathways through the olfactory route [21]. Hyperintensity of the olfactory tracts on T2 FLAIR and DWI brain MRI sequences has been observed, as has bilateral transient olfactory bulb edema in patients with anosmia related to COVID-19 [22, 23].

Headache is a frequent symptom of SARS-CoV-2 infection (31.7%), especially early in the disease (90.1%) and characterized as previously described, which would correspond to a headache attributed to systemic viral infection. In fact, headache is a common manifestation of respiratory tract viral infections [24] and was the most frequent neurological manifestation (35%) described during the H1N1 pandemic of 2009 [25]. The exact mechanism of this headache is unknown, and potential causes include fever (endogenous or exogenous pyrogens), direct effect of microorganisms, and activation by inflammatory mediators (especially relevant in SARS-CoV-2 infection) [26, 27]. In our cohort anosmia was significantly associated with headache, which suggests a direct effect of the microorganism through entry through the olfactory bulb. No obvious time relationship between hypoxia/hypercapnia and the onset of headache was observed, but it is highly likely that it also plays a role. Patients who had headache in the later stages of the disease presented with tension-type characteristics.

Encephalopathy has been described repeatedly and is linked to greater severity of SARS-CoV-2 infection and worse vital prognosis [28]. The origin of encephalopathy in these patients varies and is often multifactorial: toxic, metabolic, hypoxic-ischemic, septic, inflammatory (related to a cytokine storm with subsequent ARDS and multiple organ system failure), coagulopathy (disseminated intravascular coagulation with cerebral thromboembolic events), and possible direct involvement of the virus has also been suggested [29]. In our clinical series, the most frequent cause of encephalopathy was metabolic/hypoxic in the context of critical illness, with no relation to the virus [30]. In this sense, the need to isolate elderly patients and those with sensory deficiencies, pluripathology and frailty, makes it more likely that older patients and those with worse baseline status present with delirium.

One case that stands out is the patient with delayed awakening after mechanical ventilation. Multiple periventricular ischemic lesions were observed in both semioval centers, as well as multiple diffuse microhemorrhages in the supratentorial and infratentorial regions. Brain lesions are common in patients who require extracorporeal membrane oxygenations (ECMO): they have been described in up to 50% of cases, and hypoxic-ischemic and hemorrhagic lesions have been observed in autopsies [31]. In the H1N1 pandemic of 2009, cases of ischemic and hemorrhagic lesions were reported in patients receiving ECMO [32]. Cerebral microhemorrhages have also been described in patients with prolonged severe hypoxemia requiring mechanical ventilation: for example, in patients with ARDS, with diffuse microhemorrhages typically affecting the corpus callosum [33]. In the case of COVID-19, it has also been posited that endotheliopathy related to viral infection and thrombotic migroangiopathy [34] may contribute.

An increase in the biomarkers of CNS injury has been observed in patients with COVID-19, in association with the severity of the disease [35]. In our cohort, however, patients with the most severe illness at the time of hospitalization (CURB-65 ≥ 2 and lower SpO2/FiO2) presented with significantly less anosmia, dysgeusia, and headache. Our study only included hospitalized patients, which may mean that the prevalence of these symptoms is greater than in mild cases. It is also possible that patients with more severe illness paid less attention to these symptoms. In contrast, the absence of dyspnea is significantly more frequent in patients with more severe illness, likely due to the aforementioned pathophysiological mechanisms at the pulmonary level.

It has been hypothesized that SARS-CoV-2 infection could be a precipitating factor for stroke, since patients with severe COVID-19 present with elevated d-dimer levels, thrombocytopenia, and pathological conditions of microangiopathy [36]. The incidence of stroke reported in previous studies has been highly variable, ranging from < 2% [5, 36] to 26% [9]. It is higher than in the flu [36] and in association with greater mortality [37]. We only observed two cardioembolic strokes due to non-anticoagulated atrial fibrillation, with no relation to the virus.

In our clinical series, tremor was either due to the aggravating of pre-existing tremor (parkinsonism or essential tremor) or was secondary to medication or hypoxia. Parkinsonism has been posited as a possible neurological sequela [38, 39] but we did not observe it over the short term. Myoclonus has also been described [40], but in our findings, it was related to hypoxic, toxic, or metabolic encephalopathy.

Muscle symptoms are frequent in severe systemic viral infections and multifactorial in origin, so it is no surprise that we observed myalgia in 32.7% of patients. Of these, 95.5% presented with myalgia as an early symptom. Myopathy appeared during hospitalization or the recovery phase in 93.8% of patients who presented with it (7.8%). In the literature, 10% of patients present with myalgia and weakness with very elevated CK levels (> 10,000) [4]. These results are not replicated in our clinical series, in which there was no relationship between high CK levels and the presence of myalgia.

Aside from the cognitive involvement of any severe illness or prolonged hospitalization [11], the potential repercussions of the infection and subsequent neuroinflammation on the onset and progression of neurodegenerative and neuropsychiatric diseases have been considered [41].

COVID-19 involves an inflammatory reaction with elevated cytokine levels, which is related to the onset of cognitive impairment, especially in long-term memory [42]. Hypoxia and pharmacological treatments can also be at the root of attention deficits and processing speed.

In line with other studies [43, 44], we observed a considerable rate of patients with reduced attention, working memory, learning and retrieval, executive functions, and processing speed at three months after hospital discharge. Despite the cognitive improvement observed one year after hospital discharge, some patients still had cognitive impairment. Higher rates of abnormal cognition have been reported in other series 12 months after hospitalization for severe COVID-19 [45].

It is interesting how anosmia is predictive of increased impairment of working memory at 3 months, which is the basis of many complex cognitive functions. This, together with the greater presence of headache may suggest a direct or indirect lesion from SARS-CoV-2 at the level of the prefrontal cortex through invasion of the olfactory bulb.

38.7% of patients presented with anxiety, and 46.8% showed depressive symptoms. These high rates are similar to those observed in previous studies [46]. Isolation, low distress tolerance, and the lack of family support during hospitalization may be at the root of the mental health symptoms presented by this cohort in the first wave of the pandemic.

### Limitations

We do not aim to extrapolate our findings to the general population of patients with COVID-19. Rather, this study reflects patients with characteristics and disease severity that result in the need for hospitalization. When we conducted the study, the existing evidence recommended empirical treatment with immunomodulators and anticoagulants, which may have influenced the natural evolution of the disease and resulted in a low incidence of immunological and thrombotic phenomena in our series. Due to safety restrictions, it was not allowed to perform autopsies on these patients.

## Conclusions

Neurological manifestations are common (74,6%) in patients hospitalized for COVID-19. These generally include myalgia and headache associated with viruses, although the high prevalence of anosmia and dysgeusia suggesting CNS involvement is surprising. We have confirmed that these initial manifestations are largely resolved by follow-up at one month. Other neurological manifestations were also observed, such as encephalopathy, tremors, myoclonus, myopathy, and neuropathy. However, in most cases, they do not have a causal relationship with the virus and are related to the frailty and pluripathology of hospitalized patients. The cognitive impairment observed at 3 months in this subgroup of patients stands out. They show improvement at follow-up at 12 months, but impaired attention persists in 14.3% of cases. Patient severity is not related to an increase in neurological manifestations or to the cognitive impairments observed. Anosmia is associated with more headache and a greater risk of working memory impairment, which may suggest neurotropism with a direct or indirect SARS-CoV-2 lesion in the prefrontal cortex through invasion of the olfactory bulb.

## Electronic supplementary material

Below is the link to the electronic supplementary material.

Supplementary Material 1

Supplementary Material 2

## Data Availability

The data that support the findings of this study are available from the corresponding authors, upon reasonable request.
