# Supplementary material for: Neurologic features in hospitalized patients with COVID-19: a prospective cohort in a catalan hospital
Source: Neurol Sci. 2025 Feb 14;46(4):1477–88. doi: 10.1007/s10072-025-08031-y (PMC11920300; doi:10.1007/s10072-025-08031-y)
Supplement: Supplementary file 1 — Supplementary Material 1 [file 10072_2025_8031_MOESM1_ESM.docx]

| **Topography** | **Symptom** | **Direct or indirect** | | **Non-specific** | | | | | **Total** |
| --- | --- | --- | --- | --- | --- | --- | --- | --- | --- |
|  |  | **n (%)** | **Diagnosis** | **n (%)** | | **Diagnosis** | | |  |
| Cranial nerves | Anosmia | 51 (100) | Anosmia attributed to viral infection | 0 (0) | |  | | | 51 |
|  | Dysgeusia | 60 (100) | Dysgeusia attributed to viral infection | 0 (0) | |  | | | 60 |
|  | Vertigo | 2 (100) | Peripheral vertigo attributed to viral infection | 0 (0) | |  | | | 2 |
| Central nervous system | Headache | 59 (89.4) | Headache attributed to viral infection | 7 (10.6) | 6 | Tension | | | 66 |
|  |  |  |  |  | 1 | Migraine | | |  |
|  | Encephalopathy | 12 (26.7) | Encephalopathy attributed to viral infection | 33 (73.3) | 14 | Confusional syndrome | | | 45 |
|  |  |  |  |  | 1 | Post-extubation delirium and agitation | | |  |
|  |  |  |  |  | 1 | Encephalopathy due to post-ECMO ischemic lesions and hemorrhages | | |  |
|  |  |  |  |  | 17 | Systemic encephalopathy | 6 | Hypoxia |  |
|  |  |  |  |  |  |  | 3 | Hypercapnia |  |
|  |  |  |  |  |  |  | 3 | Hyponatremia |  |
|  |  |  |  |  |  |  | 2 | Diabetic ketoacidosis |  |
|  |  |  |  |  |  |  | 3 | Uremia |  |
|  |  |  |  |  |  |  | 2 | Hepatic |  |
|  |  |  |  |  |  |  | 1 | Digoxin toxicity |  |
|  | Cerebrovascular disease | 0 (0) |  | 3 | 2 | Cardioembolic stroke due to non-anticoagulated atrial fibrillation | | | 3 |
|  |  |  |  |  | 1 | Hemorrhagic and ischemic lesions due to ECMO | | |  |
|  | Seizures | 0 (0) |  | 1 | | Debut vascular epilepsy | | | 1 |
|  | Movement disorder | 0 (0) |  | 14 | 7 | Tremor | 1 | Worsening of essential tremor secondary to drugs or hypoxia | 14 |
|  |  |  |  |  |  |  | 2 | Worsening of parkinsonism in the context of treatment withdrawal and fever |  |
|  |  |  |  |  |  |  | 2 | Iatrogenic tremor due to corticosteroids and/or hypoxia |  |
|  |  |  |  |  |  |  | 1 | Iatrogenic tremor due to lopinavir/ritonavir |  |
|  |  |  |  |  | 7 | Myoclonus of hypoxic, toxic or metabolic origin | | |  |
|  | Vision disorder | 4 (57.1) | Blurry vision and/or myodesopia in the context of systemic viral infection | 3 (42.9) | 1 | Glaucoma | | | 7 |
|  |  |  |  |  | 1 | Epiretinal membrane | | |  |
|  |  |  |  |  | 1 | Bilateral vitreous detachment | | |  |
| Peripheral nerves and muscles | Myalgia | 64 (95.5) | Myalgia attributed to systemic viral infection | 3 (4.5) | | Myalgia in the context of recovery | | | 67 |
|  | Myopathy | 0 (0) |  | 16 | 7 | Critical illness myopathy | | | 16 |
|  |  |  |  |  | 9 | Disuse muscle atrophy | | |  |
|  | Neuropathy | 2 (20) | Acute polyneuropathy in the context of systemic viral infection | 8 (80) | 1 | Polyneuropathy of critical illness | | | 10 |
|  |  |  |  |  | 7 | Compressive neuropathies | | |  |
|  |  |  |  |  | 1 | Worsening of lumbar spinal stenosis due to immobility | | |  |
| Overall | | 254 (74.3) |  | 88 (25.7) | |  | | | 342 |

**Supplemental Table 1** Causal relationship between neurological manifestation and SARS-CoV2
