# Supplementary material for: Neurologic features in hospitalized patients with COVID-19: a prospective cohort in a catalan hospital
Source: Neurol Sci. 2025 Feb 14;46(4):1477–88. doi: 10.1007/s10072-025-08031-y (PMC11920300; doi:10.1007/s10072-025-08031-y)
Supplement: Supplementary file 2 — Supplementary Material 2 [file 10072_2025_8031_MOESM2_ESM.docx]

|  | Patients (n=62) |
| --- | --- |
| Age (years), mean (SD) | 51.32 (10.84) |
| Female (%) | 30 (48.4%) |
| Years education, mean (SD) | 10.89 (4.23) |
| IQ^a^, mean (SD) | 98.27 (9.37) |
| Hospitalization days, mean (SD) | 5.05 (4.08) |
| SpO2/FiO2, mean (SD) | 439.59 (56.60) |
| D-dimer, mean (SD) | 1612.04 (3192.93) |
| Ferritin, mean (SD) | 1276.15 (1284.69) |
| CURB-65 | |
| 0-1 (%) | 18 (29%) |
| 2 (%) | 41 (66.1%) |
| 3-5 (%) | 3 (4.8%) |
| NIMV^b^(%) | 9 (14.5%) |
| Headache (%) | 43 (69.4%) |
| Anosmia (%) | 33 (53.2%) |
| Hypogeusia (%) | 38 (61.3%) |
| Myalgia (%) | 38 (61.3%) |

**Supplemental Table 2** Descriptives and clinical frequencies of cognitive study patients ^a^IQ: Intellectual Quotient ^b^NIMV: Non-Invasive Mechanical Ventilation
